# Inhibitory Effects of Diclofenac on Steroid Glucuronidation In Vivo Do Not Affect Hair-Based Doping Tests for Stanozolol

**DOI:** 10.3390/molecules22060976

**Published:** 2017-06-12

**Authors:** Gergely Zachár, Naved I. K. Deshmukh, Andrea Petróczi, Andrea D. Székely, Iltaf Shah, James Barker, Declan P. Naughton

**Affiliations:** 1 Department of Anatomy, Histology and Embryology, Semmelweis University, Tűzoltó u. 58, Budapest 1094, Hungary; gzachar@gmail.com (G.Z.); andrea.d.szekely@gmail.com (A.D.S.); 2 School of Life Sciences, Pharmacy and Chemistry, Kingston University, Kingston upon Thames, London KT1 2EE, UK; naved1098@yahoo.co.in (N.I.K.D.); A.Petroczi@kingston.ac.uk (A.P.); J.Barker@kingston.ac.uk (J.B.); 3 Department of Chemistry, College of Science, United Arab Emirates University, Al Ain 009713, UAE; altafshah@uaeu.ac.ae

**Keywords:** steroid, metabolism, anti-inflammatory drug, inhibition

## Abstract

In vitro studies show that diclofenac inhibits enzymatic steroid glucuronidation. This study was designed to investigate the influence of diclofenac on the excretion of stanozolol and 3′-hydroxystanozolol via analyses in hair, blood and urine in vivo in a rat study. Brown Norway rats were administered with stanozolol (weeks 1–3) and diclofenac (weeks 1–6). Weekly assessment of steroid levels in hair was complemented with spot urine and serum tests. Levels of both stanozolol and 3′-hydroxystanozolol steadily increased in hair during stanozolol treatment and decreased post-treatment, but remained readily detectable for 6 weeks. In contrast, compared to control rats, diclofenac significantly reduced urinary excretion of 3′-hydroxystanozolol which was undetectable in most samples. This is the first report of diclofenac altering steroid metabolism in vivo, detrimentally affecting detection in urine, but not in hair, which holds considerable advantages over urinalysis for anti-doping tests.

## 1. Introduction

The major elimination and deactivation pathway for anabolic steroids (AS) and their phase I metabolites is through glucuronide conjugation (phase II metabolism) followed by excretion in urine [1,2,3,4]. The glucuronidation of AS is mainly catalysed by the enzyme UDP glucuronosyltransferase 2 family, polypeptide B17 (UGT2B17). Inter-individual and inter-ethnic variations in the prevalence of deletion polymorphism in the gene coding of the UGT2B17 enzyme were noted to influence the urinary excretion of AS [5,6]. Furthermore, UGTs are not only involved in the metabolism of AS, but they also contribute to the metabolism of various compounds including non-steroidal anti-inflammatory drugs (NSAIDs) [1]. The NSAIDs ibuprofen and diclofenac are commonly used by athletes for the treatment of pain and inflammation [7,8,9,10,11,12], and it has been reported that both drugs competitively inhibit the testosterone glucuronidation activity of UGT2B17 and other UGTs, in vitro [13]. Common dietary substances such as white tea and green tea [14] and red wine [15], have also exhibited similar inhibitory effects in in vitro studies. A recent study investigated the effects of two NSAIDs (ibuprofen and diclofenac) on excretion of a single dose of testosterone in 23 male volunteers with two (39%), one (33%) or no (28%) allele of the UGT2B17 gene. Intramuscular administration of the equivalent of 360 mg of testosterone along with the maximum safe dosage of NSAID had no effects on the urinary ratio of testosterone glucuronide (TG) to epitestosterone glucuronide (EG). However, this result was attributed to a slight increase in EG secretion coupled with a slight decrease in TG section. The authors also postulated that oral steroid administration may result in more marked effects owing to higher levels of metabolism via the liver [16].

Considering that such genetic and metabolic variations may limit the efficacy of urinalysis in doping tests, urinalysis, if used as a stand-alone test, is susceptible to confounding doping results [1,2,3,4,17,18]. Since impaired glucuronidation leads to reduction in the urinary excretion of AS, it can be assumed that the levels of unconjugated AS and their phase I metabolites in the systemic circulation will be elevated and thus available to be incorporated into hair and other body tissues [6]. Thus, hair analysis and blood analysis can provide complementary information to urinalysis to prevent false negative doping results. To investigate this option further, in vivo studies are required to establish a relationship between AS levels detected in hair, urine and blood.

### Aims and Research Hypotheses

With the view of addressing concerns over masking effects of NSAIDS in doping testing, this study aims to investigate the potential application of hair analysis when urinary tests are impaired. Specifically, this study was designed to investigate the influence of diclofenac on the excretion of stanozolol and 3′-hydroxystanozolol via analyses in hair, blood and urine in vivo in a rat study. Diclofenac was selected as the inhibition profiles were fully characterised in in vitro studies against UGT2B17 and UGT2B15 with diclofenac being the most potent of the NSAID inhibitors [13]. Based on in vivo studies, we expected that concomitant administration of diclofenac with stanozolol will:

H1: increase the circulating concentration of stanozolol and 3′-hydroxystanozolol (blood);
H2: decrease the concentration of 3′-hydroxystanozolol in urine;
H3: increase the concentration of stanozolol and 3′-hydroxystanozolol in hair; and
H4: whilst the concentrations of stanozolol and 3′-hydroxystanozolol in hair are expected to gradually decrease after ceasing stanolozol treatment, both stanozolol and 3′-hydroxystanozolol in hair will remain detectable for the same period after the treatment cycle.

**Figure 1:**
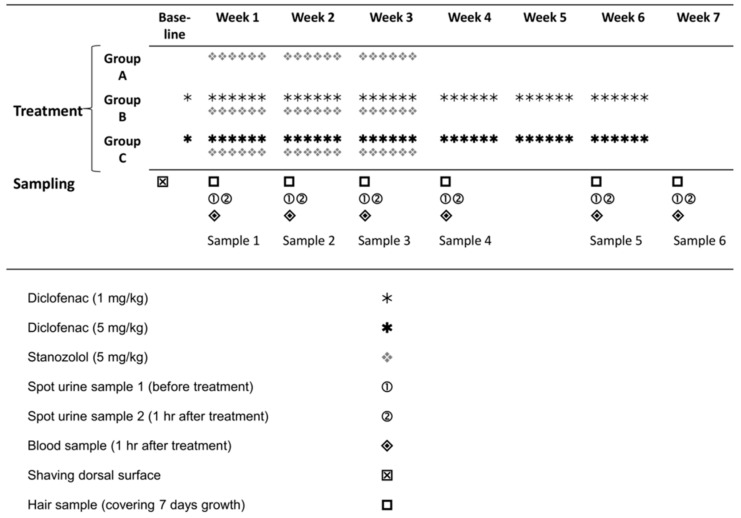
Drug treatment and sample collection period.

The study design involved administration of stanozolol to a control group and two treatment groups which received diclofenac (Figure 1) with sampling of hair, blood and urine as indicated for analysis of parent steroid and its hydroxylated metabolite.

## 2. Results

### 2.1. Effect of Diclofenac on the Serum Concentration of Stanozolol and 3′-Hydroxystanozolol

**Figure 2:**
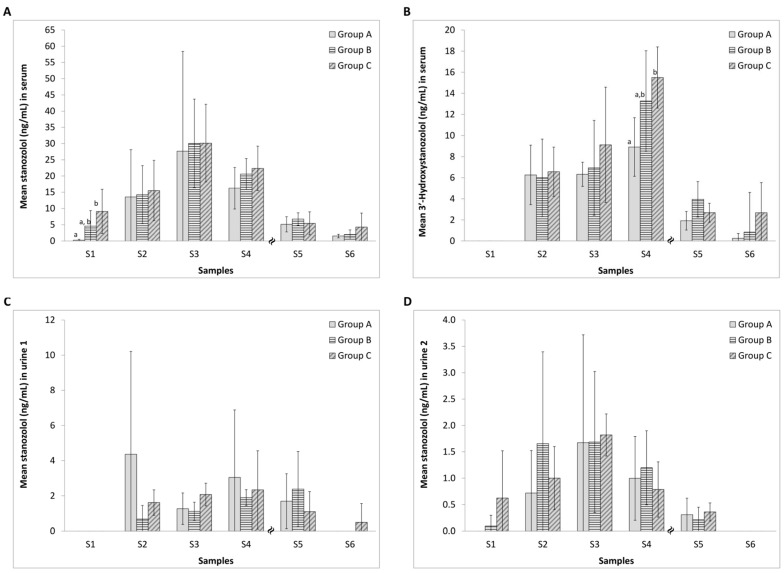
Concentrations of stanozolol and 3′-hydroxystanozolol in serum ((**A**,**B**) respectively) and stanozolol in urine samples ((**C**) pre-treatment (urine 1) and (**D**) one hour post-treatment (urine 2)) from group A (5 mg/kg stanozolol only), group B (5 mg/kg stanozolol + 1 mg/kg diclofenac) and group C (5 mg/kg stanozolol + 5 mg/kg diclofenac). Error bars represent standard deviations. Different letters (A,B) denote statistically significant differences between the treatment groups (*p* < 0.05) at a sampling point. Effect sizes are given in Table S1. Test sensitivity over time differences, are displayed in Table S2.

The concentrations of stanozolol and 3′-hydroxystanozolol in rat serum during the seven weeks of the study are shown in Figure 2A,B. As expected, mean levels of stanozolol increased during the three treatment weeks for all three groups (to ca. 30 ng/mL) with a steady decrease in levels in the subsequent four weeks post-treatment (Figure 2A). Statistical analysis reveals significant differences with diclofenac treatment for S1 and a large partial η^2^ (ABC) effect size for S1 (Table S1). Large Cohen’s d effect sizes are also revealed for S1 (A-B; A-C), S4 (A-C) and S6 (A-C; B-C) (Table S1). Test sensitivity data are in the range of 93–100% for S2–S5 (Table S2) suggesting that stanozolol and its key metabolite were detected in most samples with only one false negative result.

As expected, relative to the parent drug, lower sera levels of 3′-hydroxystanozolol were found with an increase over weeks S2–S4 (to ca. 15 ng/mL) with a steady decrease in levels in subsequent weeks post-treatment. Significant differences upon treatment with diclofenac appeared at week S4 and a large partial η^2^ (ABC) effect size occurred at S4 and S5 (Table S1). Large Cohen’s d effect sizes are found at S4 and S5 (Table S1) with test sensitivity between 78–100% for S2–S5 (Table S2). Effect sizes are used to identify apparent differences in the figure which fail to reach statistical significance but are likely to be present and practically meaningful. Failing to reach statistical significance in such cases are the combined function of the small sample size and relatively large SD. The metabolite, 3′-hydroxystanozolol, was not detectable in any of the first blood samples that were collected one hour after the administration of drugs.

### 2.2. Effect of Diclofenac on the Urinary Excretion of Stanozolol and 3′-Hydroxystanozolol

The average concentrations of stanozolol in rat urine 1 (30 min post anaesthesia) and urine 2 (60 min post drug injection) in each group during stanozolol treatment and post-stanozolol treatment are presented in Figure 2C,D. Urinalysis revealed increasing levels of stanozolol in urine 2 samples as treatment progressed with a decrease after cessation of stanozolol administration. Administration of diclofenac resulted in a large partial η^2^ (ABC) effect size observed for S3 (ABC) and large Cohen’s effect sizes occur for S2, S3 and S6 (Table S1). Sensitivity test data for stanozolol were high (94–100%) over S2–S5 (Table S2) showing only 1 false negative result.

3′-Hydroxystanozolol data in urine samples are not depicted because the metabolite was only detected in urine 1 samples in Group A in S2 (mean concentration for *n* = 6 was 8.241 ± 7.212 ng/mL); and at a low level in S3 in 4 animals of the 6 (mean concentrations = 0.188 ± 0.147 ng/mL (*n* = 4)). In S3, 3′-hydroxystanozolol was detected in urine 1 samples from one animal in each Group B (0.056 ng/mL) and Group C (0.076 ng/mL). 3′-hydroxystanozolol was not detected in any of the urine 2 samples. For 3′-hydroxystanozolol in urine 1, diclofenac administration resulted in large effect sizes being observed at S2 and S3 (Cohen’s d and partial η^2^), with significant differences at S2 (Table S1). Sensitivity test data for 3′-hydroxystanozolol in urine 1 and 2 samples were very low between 0–36% for S2–S5, reflecting a high number of false negatives (Table S2).

### 2.3. Effect of Diclofenac on the Incorporation of Stanozolol and 3′-Hydroxystanozolol in Hair

**Figure 3:**
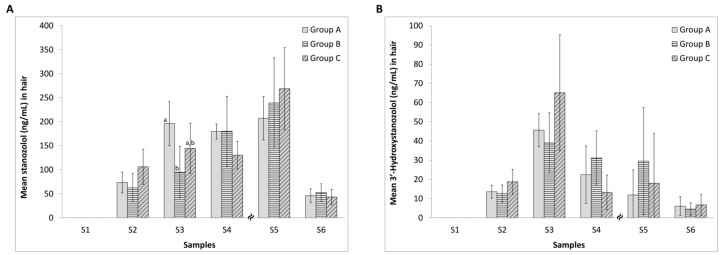
Concentrations of stanozolol (**A**) and 3′-hydroxystanozolol (**B**) in hair samples from group A (5 mg/kg stanozolol only), group B (5 mg/kg stanozolol + 1 mg/kg diclofenac) and group C (5 mg/kg stanozolol + 5 mg/kg diclofenac). Error bars represent standard deviations. Different letters denote statistically significant differences between the treatment groups (*p* < 0.05). Effect sizes are given in Table S1.

The concentrations of 3′-hydroxystanozolol and stanozolol in rat hair during the seven weeks of the study are presented in Figure 3. During the stanozolol treatment period, elevations in hair levels of stanozolol and 3′-hydroxystanozolol were observed. In all three groups, the maximum concentration of stanozolol in hair was reached on the 6th week of the study, i.e., three weeks after stopping the stanozolol treatment. However, the peak concentration of 3′-hydroxystanozolol in hair in all three groups was reached on the 3rd week of the study, i.e., after two weeks of stanozolol treatment, and then decreased gradually.

After cessation of stanozolol treatment, the levels decline at S6 for parent drug and S4 onwards for the metabolite. There were significant differences owing to diclofenac at S3 for the parent drug reflecting a decrease in hair uptake. For the stanozolol levels, diclofenac treatment resulted in the partial η^2^ effect sizes being large at S2 and S3 with significant difference at S3 and with large values for Cohen’s d effect size at S2–S5 (Table S1). The test sensitivity is between 93–100% across S2–S6 (Table S2). During the latter weeks, levels of stanozolol in hair were elevated up to S5, and then decreased, but still present in abundance, in all three groups. For 3′-hydroxystanozolol, large partial η^2^ effect sizes are seen for S2–S4 and Cohen’s effect sizes for S2–S5 reflecting higher levels of metabolite in hair, especially for the group receiving 1 mg/kg diclofenac with the stanozolol treatment.

### 2.4. Relationship between Levels of Stanozolol and 3′-Hydroxystanozolol in Hair, Urine and Blood

Table 1 displays the relationships between hair levels of stanozolol and 3′-hydroxystanozolol and levels in urine and blood two weeks prior to the hair sample collection, separately for each experimental group. During the stanozolol treatment period (S1–S3), a relationship (*r* > 0.3) with urine levels of stanozolol and 3′-hydroxystanozolol was only observed in the two groups that also received diclofenac. In Group A, hair levels after the treatment period showed a positive relationship with levels in blood. In groups B and C, no clear pattern is observed, however, it was notable that several positive relationships between levels in S3 (last treatment week) in body fluids and two weeks later in hair (S5) turned negative one week later. The inverse pattern is also observable in Figure 2 and Figure 3, where in hair, stanozolol concentration—at the group level—peaked at S5 (2 weeks after the stanozolol treatment ceased), whereas the metabolite level peaked at S3 (end of stanozolol treatment). The pattern was reversed for blood, albeit less pronounced, where stanozolol concentration peaked at S3 whereas the 3′-hydroxystanozolol level was the highest in all 3 groups at S4.

## 3. Discussion

### 3.1. Effect of Diclofenac on Stanozolol Distribution

#### 3.1.1. H1: Increase the Circulating Concentration of Stanozolol and 3′-Hydroxystanozolol (Blood)

The experimental design featured the capability of assessing the effects of diclofenac on stanozolol profiles during co-administration and for a four week period after cessation of stanozolol to capture the effect on extended metabolism. Diclofenac administration increased the circulating levels of stanozolol and 3′-hydroxystanozolol (H1) as assessed by significant differences and/or large effects sizes for the period S1, S4 and S6 for stanozolol and S3–S6 for 3′-hydroxystanozolol. The effect is notable on the levels of metabolite over the final weeks (S4–S6). This disruptive effect on metabolism is in line with inhibition of glucuronidation by diclofenac, whereby a build-up of 3′-hydroxystanozolol occurs. The early phase significant difference for stanozolol levels may result from a failure to clear the metabolite owing to impaired glucuronidation. However, the low levels of metabolite at S1 (below the LLOQ) point to another explanation such as impaired phase 1 metabolism affecting generation of the hydroxylated metabolite.

#### 3.1.2. H2: Decrease the Concentration of 3′-Hydroxystanozolol in Urine

Urinalysis results followed an expected pattern in that levels of 3′-hydroxystanozolol were below the limit of quantification in all but 2 of the diclofenac treated rat samples (out of a total of 144 samples taken) despite the assay design to measure combined free metabolite and de-glucuronidated metabolite in line with testing protocols for stanozolol [19,20]. In contrast, 3′-hydroxystanozolol was readily detectable in most of the samples taken at S2 (mean concentration 8.241–7.212 ng/mL) and S3 from rats without diclofenac treatment. Low quantities of stanozolol were measured in both urine 1 and 2 samples, which highlighted the absence of quantifiable levels of the metabolite or its glucuronide conjugate. In summary, administration of diclofenac decreased the urinary levels of 3′-hydroxystanozolol to below the limit of quantification in all except 1 rat at S3 for both doses of diclofenac. This outcome is supported by large effect sizes at S2 and S3, with significant differences at S2 (Table 1).

#### 3.1.3. H3: Increase the Concentration of Stanozolol and 3′-Hydroxystanozolol in Hair

After one week of stanozolol treatment, very high levels of the parent drug and its metabolite (relative to the LLOQ; 0.5 pg/mg) were measured in hair samples in all sampling weeks S2–S6. These levels of up to 600× and 120× the LLOQ for stanozolol and 3′-hydroxystanozolol respectively are present in every sample taken after week one of treatment. Treatment with diclofenac resulted in an initial decrease in hair stanozolol levels at week S3. An analogous variable picture is observed for 3′-hydrozystanozolol where diclofenac treatment resulted in higher levels at S3–S5 for at least one diclofenac dose regime. Thus, diclofenac has variable effects on hair uptake of stanozolol, but increased levels are seen after two weeks of administration. This increase is off-set for 3′-hydroxystanozolol until week S4; that is three weeks after initiation of treatment. Thus, variable effects are observed for diclofenac on stanozolol and 3′-hydroxystanozolol levels in hair. In each case, the levels present for parent drug and metabolite greatly exceed the LLOQ. This variability may arise from the effects of large doses of diclofenac coupled to the complex uptake modes into hair which depend on numerous factors ranging from levels and activity of UGT enzymes, to the basicity of the analyte itself.

#### 3.1.4. H4: Whilst the Concentrations of Stanozolol and 3′-Hydroxystanozolol in Hair are Expected to Gradually Decrease after Ceasing Stanolozol Treatment, Both Stanozolol and 3-Hydroxystanozolol in Hair Will Remain Detectable for up to Three Weeks

The levels of stanozolol in hair had started to decline at week seven, a full three weeks after administration stopped. The maximum levels were observed in week S5 at some 600 times the LLOQ. For week seven, the levels had dropped considerably but were still some ten times the LLOQ. Unfortunately, the available data are not sufficient to estimate how long the parent drug stanozolol still remains detectable in hair, but extrapolating the rate of decline, future studies should continue with weekly sampling up to 6 weeks (twice the treatment period) after the treatment ceased. In contrast, the levels of 3′-hydroxystanozolol reached a peak at week S3 and declined thereafter. Although they had diminished, the levels of the metabolite in hair were still considerably above the LLOQ at week S6. The overall results from the hair analysis suggest that diclofenac did not inhibit the incorporation of stanozolol or 3′-hydroxystanozolol into hair. Following cessation of stanozolol treatment, both compounds were detected in hair in all three groups up to four weeks after the administration of the last dose of stanozolol whilst diclofenac administration continued.

### 3.2. Implications of the Results and Future Perspectives

The results are of particular interest to those involved in anti-doping tests. One key finding is that hair analysis is far superior to urinalysis for the out of competition detection of synthetic steroids as part of an anti-doping test regime. In spite of three weeks of continuous daily dosing (for 6 days per week), levels of the steroid metabolite 3′-hydroxystanozolol in urine were below the limit of quantification for this assay, whereas for the same dosage, the levels in hair were over one hundred-fold above the level of quantification. A further concern for doping control is that the NSAID led to reduced levels of metabolite in urine leading to potential false negative test results. On the contrary, hair was unaffected. The difficulties of utilising urinalyses for stanozolol based on quantification of the hydroxylated metabolite are seen with the sensitivity results showing a very high proportion of false negatives (Table S2). In contrast, for the parent steroid, levels in hair are over 200-fold above the LLOQ with detection being feasible for up to three weeks after cessation of the steroid administration. Although the study was conducted in rats, the method used provided a limit of detection below test criteria for stanozolol and 3′-hydroxystanozolol used by the World Anti-Doping Agency (WADA) stablished measure of Minimum Required Performance Levels (2 ng/mL) (WADA TD2014MRPL).

In line with the standard doping testing protocol, the test method used for hair incorporated both 3′-hydroxystanozolol and the de-glucuronidated conjugate [20]. In addition to the capability of a single test covering several months (as 1 cm of cranial hair growth equates to ca. 1 month), hair analysis provides a number of advantages over urine or serum testing. The known advantages prior to the study and presented in this paper are catalogued as reduced infection risk, non-invasive sample collection, facile sample storage and transport, reduced risk of tampering and ease of chain of custody. Further advantages are that the test does not rely on knowing the location of an athlete or having them available for testing on a regular basis. In support of hair-based testing, this study provides strong evidence that detecting a synthetic steroid in hair is unaffected by temporary compromised metabolism via concomitant administration of NSAID. Although further research is warranted, including human studies, the results demonstrate, for the first time in vivo, that diclofenac, an NSAID interacts with steroid metabolism, a long suggested pathway based on in vitro studies. The results indicate that diclofenac significantly inhibits the urinary excretion of 3′-hydroxystanozolol in rats. However, diclofenac was found to have no inhibitory effect on the incorporation of stanozolol and 3′-hydroxystanozolol in hair. Even after ending stanozolol treatment, stanozolol was detectable in the hair of most animals after three weeks. In contrast, stanozolol was detectable in the urine of only two animals (out of 17) three weeks after cessation of stanozolol treatment, whereas 3′-hydroxystanozolol was not detectable in the urine samples of any animals. These results highlight the difficulties of the detecting both compounds in urine in comparison to hair analyses. Blood analysis can also provide important information on drug use, but it is a short term measure and an invasive method compared to hair.

It is envisaged that results from this study can be extended to other drugs in the synthetic steroids family and move towards developing hair-based testing for a wider range of synthetic steroids as a part of new doping control measures in competitive sport. In the past, researchers have found it difficult to detect 3′-hydroxystanozolol in hair. Cirimele et al. reported the detection of stanozolol in scalp hair of a bodybuilder who declared regular use of stanozolol [21]. However, 3′-hydroxystanozolol was not detectable in hair under the analytical conditions employed by the authors. Similarly, in another study carried out by Shen et al. stanozolol was detectable in guinea pig hair after administering stanozolol at a single high dose of 60 mg/kg, whereas, 3′-hydroxystanozolol was not detectable [22]. However, the method developed for the current study is capable of detecting stanozolol and 3′-hydroxystanozolol in rat hair after administering stanozolol for 6 days at a dose of 5.0 mg/kg/day that is considered equivalent to those levels abused by athletes [6].

## 4. Materials and Methods

### 4.1. Chemicals and Reagents

Reference standards for stanozolol, 3′-hydroxystanozolol, 3′-hydroxystanozolol glucuronide, stanozolol D3, 3′-hydroxystanozolol D3 were purchased from LGC standards (Teddington, UK). All other chemicals were purchased from Sigma Aldrich (Poole, UK). β-Glucuronidase from *E. coli* was purchased from Roche Diagnostics (Burgess Hill, UK). For the animal experiments, stanozolol, ketamine (2.5%), xylazine (Rompun, 2%) and diclofenac were purchased from Desma (Madrid, Spain), Kőbányai Gyógyszerárugyár (Budapest, Hungary), Haver-Lockhart Laboratories (Shawnee, KS, USA) and Sigma Aldrich (Deisenhofen, Germany) respectively.

### 4.2. Animals

Eighteen male, Brown Norway rats were purchased from Charles River laboratories (Sulzfeld, Germany). Each animal weighed around 280–340 g and was approximately 5 months old. Rats were housed in groups of three individuals in standard laboratory cages and kept in a constant room temperature environment with an alternating 12-h light-dark cycle. Food and water were available ad-libitum. The administration of drugs and sample collection were conducted under the institutional license of the Department of Anatomy, Histology and Embryology, Semmelweis University (Budapest, Hungary) in accordance with the EC Council directives on laboratory animals (86/609/EEC) [23].

### 4.3. Administration of Diclofenac and Stanozolol

Rats were divided into three groups of six animals each (Groups A, B and C). Diclofenac, dissolved in saline, was administered daily, subcutaneously, to each animal belonging to group B and C, six days a week at doses detailed in Figure 1. Two days after the beginning of diclofenac treatment, animals of all three groups received stanozolol (in saline) intraperitoneally [22], at a dose of 5 mg/bmkg/day, six days a week. The diclofenac treatment lasted for a period of six weeks, whereas the stanozolol treatment lasted for three weeks (Figure 1). Stanozolol and diclofenac were injected at the same time on every treatment day from Monday to Saturday (inclusive).

Group B received diclofenac at a daily dosage of 1 mg/bmkg and the recommended daily dose of diclofenac in adults is 75 to 150 mg daily. Thus, depending on the body weight of adult individuals, dosage of diclofenac received by Group B approximately resembled the typical daily dose of diclofenac in adults. The dose of stanozolol selected was in line with previous steroid studies using rat models and considered equivalent to those abused by humans on a milligram per kilogram of body weight basis [24,25,26,27]. Group C received a dose of 5 mg/bmkg to ascertain the effects of high dosage on steroid distribution. Negative controls were obtained by collecting hair, blood and urine samples prior to the administration of the drugs to the rats.

### 4.4. Sample Collection

The hair segments, blood and two urine samples were collected under anaesthesia (ketamine and xylasine mixture) once every week for five weeks and at week seven (Figure 1). Sample collections were scheduled for Wednesdays (after two days of the weekly treatment cycle) in all samples but S2, where sample collection took place on Tuesday (on the second day of the weekly treatment cycle).

At sample collections, each animal was weighed and hair samples were collected during the first 30 min after anaesthesia by shaving an area on the back of animals using an electric shaver. Exactly the same dorsal surface was sampled each time to avoid any diluting effect of hair regrowth before the stanazolol treatment period. Urine was collected by gently pressing the abdomen. The first urine sample (urine 1) was collected 30 min after anaesthesia. The second urine (urine 2) sample was collected after one hour of stanozolol and diclofenac injections. Blood samples were taken (along with the urine 2 samples) from the tail vein one hour after stanozolol and diclofenac injection.

### 4.5. Sample Preparation

Serum and urine samples were stored at −80 °C. The LC-MS/MS methods developed for stanozolol and its metabolite have been described previously [28]. Briefly, serum and urine samples were thawed and vortexed with 100 μL used for analyses. Hair samples (50 mg) were decontaminated with dichloromethane followed by sectioning into ca. 1 mm segments prior to incubation with 1 mL of 1 M sodium hydroxide (with the internal standard (I.S.) stanozolol D3) at 95 °C for 10 min. Hydrochloric acid (1 M) was added to neutralise the homogenate, followed by addition of 2 mL of 0.2 M phosphate buffer (pH 7.0), before a liquid-liquid extraction (LLE) step using 3.5 mL pentane [28]. Enzyme digestion was utilised to determine the total concentration (glucuronide conjugated + unconjugated) of stanozolol and 3′-hydroxystanozolol in each matrix (hair, urine and serum) [28]. The methods were fully validated and lower limits of quantification (LLOQ) for stanozolol and 3′-hydroxystanozolol were calculated to be 0.125 ng/mL and 0.25 ng/mL in urine respectively, in hair 0.5 pg/mg for both analytes (using ca. 50 mg hair samples) and 0.25 ng/mL for both analytes in serum. The lower limits of detection (LLOD) for stanozolol and 3′-hydroxystanozolol were 0.063 ng/mL and 0.125 ng/mL in urine and serum, respectively. The LLOD in hair for stanozolol and 3′-hydroxystanozolol were 0.125 pg/mg and 0.25 pg/mg respectively, when ca. 50 mg hair was processed.

### 4.6. Statistical Analysis

For data analyses, levels below the LLOQ were treated as zero. The key research hypothesis, namely the effect of diclofenac on detectability of stanozolol and/or its metabolite in different matrices, was investigated with the sensitivity of the analytical tests for each matrix using the proportion of false negatives. Group differences in stanozolol and 3′-hydroxystanozolol levels were tested at each sampling point using mixed model ANOVA with single main effect test. To establish the magnitude of the difference between groups, the effect size was also calculated and expressed as partial eta-squared. Based on the study design that uses only one independent variable (treatment groups), eta-squared values can be used to interpret effect sizes expressed as partial eta-square, thus values >0.02 were considered a small effect, >0.13 were considered a medium effect, and >0.26 were considered a large effect [29].

For a detailed analysis of the magnitude of the changes as the study progressed, average treatment group levels of stanozolol and 3′-hydroxystanozolol from each sampling point were compared to similar levels from the previous week using paired sample *t*-tests. Effect sizes for the paired samples are expressed as Cohen’s d (small effect >0.2; medium effect >0.5; large effect >0.8), corrected for dependency using Morris and DeShon’s equation 8 [30]. Owing to the large variations in stanozolol and 3’-hydroxystanozolol within each group, coupled with the small sample size, changes over time in the measured levels are interpreted based on effect size and not statistical significance. This approach is in line with the growing trend of using effect size measures instead of statistical significance to allow practically meaningful interpretations [31,32].

The relationship between hair levels of stanozolol and 3′-hydroxystanozolol and in urine and blood was tentatively investigated by calculating correlation coefficients (Pearson’s r) as follows: levels in hair at each measurement point (S3–S6) was correlated with levels measured two weeks prior to taking the hair sample in question, separately for each experimental group. Two week intervals were selected based on the results showing that both stanozolol and 3′-hydroxystanozolol appeared in hair in S2, 2 weeks after the treatment commenced. Owing to the small sample size, correlation coefficients were limited to describe the strength and direction of the observed. The level of significance was set at *p* < 0.05 for all analyses. All statistical analyses were performed using SPSS 21.0 (SPSS Inc., Chicago, IL, USA).

## 5. Conclusions

An undisputable key result from this study is that following in vivo administration, stanozolol and its metabolite 3′-hydroxystanozolol are quantifiable in hair samples at levels well above the limit of quantification, and this is starkly in contrast to urinalysis, which appeared to be ineffective. This marked contrast to urinalysis suggests that hair based analyses is a strong contender for future doping tests, specifically for out of competition doping control, for synthetic steroids. A second novel outcome is that the NSAID diclofenac has significant effects on the distribution of stanozolol in vivo, with a reduction in urine levels to below the Minimum Required Performance Levels set by WADA. Further research is warranted to fully develop this clear potential as a new anti-doping test for synthetic steroids.

## Figures and Tables

**Table 1:** Relationship between levels of stanozolol and 3′-hydroxystanozolol in hair and in urine and blood samples for the closest corresponding time period (2 weeks).

		Stanozolol Treatment		Post Stanozolol Treatment		
		Weeks 1 & 2	Week 3	Week 4	Week 5	Week 6
		**Hair Stanozolol**				
	Urine 1ST	-		S2 (−) **	S3 (−) ***	
	Urine 2ST	-			S3 (−) *	
	Serum ST	-	S1 (+) **	S2 (+) *	S3 (+) *	S4 (+) *
Group A		**Hair 3′-Hydroxystanozolol**				
	Urine 1HST	-		S2 (−) *	S3 (−) ***	
	Urine 2HST	-				
	Serum HST	-		S2 (−) *	S3 (+) *	S4 (+) *
		**Hair Stanozolol**				
	Urine 1ST	-		S2 (+) ***		S4 (−) *
	Urine 2ST	-		S2 (−) **	S3 (−) *	
	Serum ST	-	S1 (−) ***		S3 (−) *	S4 (−) **
Group B		**Hair 3′-Hydroxystanozolol**				
	Urine 1HST	-			S3 (+) ***	
	Urine 2HST	-				
	Serum HST	-		S2 (+) ***	S3 (-) *	S4 (+) ***
		**Hair Stanozolol**				
	Urine 1ST	-		S2 (−) *	S3 (+) ***	S4 (−) **
	Urine 2ST	-		S2 (−) *	S3 (+) *	
	Serum ST	-	S1 (−) *	S2 (+) ***	S3 (+) ***	S4 (−) **
Group C		**Hair 3′-Hydroxystanozolol**				
	Urine 1HST	-			S3 (+) ***	
	Urine 2HST	-				
	Serum HST	-		S2 (−) *	S3 (+) **	S4 (−) *

S = sampling points; ST = stanozolol; HST = 3′-hydroxystanozolol; * = 0.3 < *r* > 0.5; ** = 0.5 < *r* > 0.7; *** = *r* > 0.7. Not shown (cell is empty) if *r* < 0.3; (−) denotes negative correlation; (+) denotes positive correlation.

## Supplementary Materials

Supplementary materials are available online. Table S1: Effect sizes for group differences at each sampling point, Table S2: Test Sensitivity.
