# Supplementary material for: Inhibitory Effects of Diclofenac on Steroid Glucuronidation In Vivo Do Not Affect Hair-Based Doping Tests for Stanozolol
Source: Molecules. 2017 Jun 12;22(6):976. doi: 10.3390/molecules22060976 (PMC6152663; doi:10.3390/molecules22060976)
Supplement: Supplementary file 1 [file molecules-22-00976-s001.pdf]

Table S1. Effect sizes[a] for group differences at each sampling point

Sample	Groups	Hair		Serum		Urine 1		Urine 2	
		Stanozolol	3’Hydroxy-stanozolol	Stanozolol	3’Hydroxy-stanozolol	Stanozolol	3’Hydroxy-stanozolol	Stanozolol	3’Hydroxy-stanozolol
S1	partial η^2 (A,B,C)	BLLOQ	BLLOQ	0.409*	BLLOQ	BLLOQ	BLLOQ	0.242	BLLOQ
	d (A-B)	-	-	1.720				-	
	d (A-C)	-	-	2.513*	BLLOQ	BLLOQ	BLLOQ	-	BLLOQ
	d (B-C)	-	-	0.785				0.923	
S2	partial η^2 (A,B,C)	0.319	0.263	0.006	0.007	0.191	0.503*	0.126	BLLOQ
	d (A-B)	0.434	0.243	0.061	0.084	1.114	2.285*	0.737	-
	d (A-C)	1.123	1.046	0.165	0.118	0.836	2.285*	0.333	-
	d (B-C)	1.326	1.114	0.138	0.192	1.255	-	0.495	-
S3	partial η^2 (A,B,C)	0.442*	0.261	0.004	0.093	0.296	0.146	0.003	BLLOQ
	d (A-B)	2.041*	0.549	0.108	0.217	0.212	0.971	0.007	-
	d (A-C)	1.060	1.002	0.117	0.844	1.045	0.675	0.119	-
	d (B-C)	0.935	1.138	0.008	0.437	1.639	0.129	0.154	-
S4	partial η^2 (A,B,C)	0.238	0.285	0.182	0.439*	0.036	BLLOQ	0.066	BLLOQ
	d (A-B)	0.013	0.598	0.777	1.155	0.536	-	0.267	-
	d (A-C)	2.222	0.769	0.925	2.303*	0.234	-	0.320	-
	d (B-C)	0.984	1.546	0.305	0.569	0.330	-	0.670	-
S5	partial η^2 (A,B,C)	0.121	0.115	0.081	0.374	0.109	BLLOQ	0.067	BLLOQ
	d (A-B)	0.456	0.861	0.775	1.587	0.370	-	0.355	-
	d (A-C)	0.938	0.310	0.110	0.865	0.443	-	0.215	-
	d (B-C)	0.320	0.431	0.488	0.982	0.783	-	0.733	-
S6	partial η^2 (A,B,C)	0.056	0.036	0.207	0.202	0.139	BLLOQ	BLLOQ	BLLOQ
	d (A-B)	0.383	0.377	0.461	1.003	-	-	-	-
	d (A-C)	0.185	0.119	1.139	1.145	0.910	-	-	-
	d (B-C)	0.523	0.494	0.810	0.832	0.910	-	-	-

[a] Partial eta squared (η^2) was calculated by SPSS. Cohen’s $|d|$ were calculated from group means and their respective SD. * denotes statistically significant difference. Group A: 5 mg/kg stanozolol only, Group B: 5 mg/kg stanozolol + 1 mg/kg diclofenac, Group C: 5 mg/kg stanozolol + 5 mg/kg diclofenac. BLLOQ: below lower limit of quantification, which was found to be 0.125 ng/mL for stanozolol and 0.25 ng/mL for 3'-hydroxystanozolol in urine; and 0.5 pg/mg for both analytes in hair, and 0.25 ng/mL for both analytes in serum.

Table S2. Test sensitivity

			S1	S2	S3	S4	S5	S6
Hair	Stanozolol	Group A	6/6	0/6	0/6	1/5	0/6	0/6
		Group B	6/6	0/5	0/5	0/5	0/5	0/4
		Group C	6/6	0/6	0/6	0/5	0/5	0/6
	Test sensitivity		0%	100%	100%	93.3%	100%	100%
			(0.0% - 18.7%)	(81.3% - 100.0%)	(81.3% - 100.0%)	(68.0% - 99.0%)	(79.2% - 100.0%)	(75.7% - 100.0%)
	3'hydroxystanozolol	Group A	6/6	0/6	0/6	1/5	3/6	2/6
		Group B	6/6	0/4	0/5	0/5	1/5	1/4
		Group C	6/6	0/6	0/6	0/5	3/5	2/6
	Test sensitivity		0%	100%	100%	93.3%	56.2%	68.8%
			(0.0% - 18.7%)	(75.7% - 100.0%)	(80.3% - 100.0%)	(68.0% - 99.0%)	(29.9% - 80.2%)	(35.12% - 87.1%)
Serum	Stanozolol	Group A	3/6	0/6	0/5	0/6	0/5	0/3
		Group B	0/6	0/5	0/5	0/5	0/5	0/2
		Group C	0/6	0/5	0/6	0/6	1/5	0/4
	Test sensitivity		83.3%	100%	100%	100%	93.3%	100%
			(58.6% - 96.2%)	(79.2%- 100.0%)	(79.2%- 100.0%)	(78.0% - 100.0%)	(68.0 % - 99.0%)	(66.2% - 100.0%)
	3'hydroxystanozolol	Group A	6/6	0/6	0/5	0/6	0/5	2/3
		Group B	6/6	0/5	0/5	0/5	0/5	0/2
		Group C	6/6	0/5	0/6	0/6	0/5	0/4
	Test sensitivity		0%	100%	100%	100%	100%	77.8%
			(0.0% - 18.7%)	(79.2%- 100.0%)	(79.2%- 100.0%)	(80.3% - 100.0%)	(78.0 - 100.0%)	(40.1% - 96.5%)
Urine 1	Stanozolol	Group A	6/6	0/6	0/6	0/6	0/6	6/6
		Group B	5/5	1/5	0/5	0/5	0/5	5/5
		Group C	6/6	0/6	0/6	0/6	0/6	4/6
	Test sensitivity		0%	94.1%	100%	100%	100%	11.8%
			(0.0% to 19.7%)	(71.2% - 99.0%)	(80.3% - 100.0%)	(80.3% - 100.0%)	(80.3% - 100.0%)	(1.8% - 36.5%)
	3'hydroxystanozolol	Group A	6/6	0/6	2/6	6/6	6/6	6/6
		Group B	5/5	5/5	4/5	5/5	5/5	5/5
		Group C	6/6	6/6	5/6	6/6	6/6	6/6
	Test sensitivity		0%	35.3%	35.3%	0%	0%	0%
			(0.0% to 19.7%)	(14.3% - 61.6%)	(14.3% - 61.6%)	(0.0% to 19.7%)	(0.0% to 19.7%)	(0.0% to 19.7%)

Table S2. *Cont.*

Urine 2	Stanozolol	Group A	6/6	0/6	1/6	0/6	2/6	6/6
		Group B	4/5	0/5	0/5	0/5	2/5	5/5
		Group C	3/6	0/6	0/6	0/6	1/6	6/6
	Test sensitivity		23.5% (6.7% - 49.9%)	100% (80.3% - 100.0%)	94.1% (71.2% - 99.0%)	100% (80.3% - 100.0%)	70.6% (44.0% - 89.6%)	0% (0.0% to 19.7%)
	3'hydroxystanozolol	Group A	6/6	6/6	6/6	6/6	6/6	6/6
		Group B	5/5	5/5	5/5	5/5	5/5	5/5
		Group C	6/6	6/6	6/6	6/6	6/6	6/6
	Test sensitivity		0% (0.0% to 19.7%)	0% (0.0% to 19.7%)	0% (0.0% to 19.7%)	0% (0.0% to 19.7%)	0% (0.0% to 19.7%)	0% (0.0% to 19.7%)

Test sensitivity is calculated for each sampling point for the pooled samples as true positives / (true positives + false negatives); 96%CI displayed in brackets. The ratio in each cell shows the number of false negatives over the total number of positive samples
